# Supplementary material for: Enhanced C/EBPβ function promotes hyperplastic versus hypertrophic fat tissue growth and prevents steatosis in response to high-fat diet feeding
Source: eLife. 2022 Apr 22;11:e62625. doi: 10.7554/eLife.62625 (PMC9071262; doi:10.7554/eLife.62625)
Supplement: Transparent reporting form [file elife-62625-transrepform1.docx]

***eLife’s* transparent reporting form**

We encourage authors to provide detailed information *within their submission* to facilitate the interpretation and replication of experiments. Authors can upload supporting documentation to indicate the use of appropriate reporting guidelines for health-related research (see [EQUATOR Network](http://www.equator-network.org/%20)), life science research (see the [BioSharing Information Resource](https://biosharing.org/)), or the [ARRIVE guidelines](http://www.plosbiology.org/article/info:doi/10.1371/journal.pbio.1000412) for reporting work involving animal research. Where applicable, authors should refer to any relevant reporting standards documents in this form.

If you have any questions, please consult our Journal Policies and/or contact us: [editorial@elifesciences.org](mailto:editorial@elifesciences.org).

**Sample-size estimation**

- You should state whether an appropriate sample size was computed when the study was being designed
- You should state the statistical method of sample size computation and any required assumptions
- If no explicit power analysis was used, you should describe how you decided what sample (replicate) size (number) to use

Please outline where this information can be found within the submission (e.g., sections or figure legends), or explain why this information doesn’t apply to your submission:

No explicit power analysis was performed. The sample size was based on previous experience with the assays used. The information about the used sample sizes can be found in the figure legend of each main and supplementary figure.

**Replicates**

- You should report how often each experiment was performed
- You should include a definition of biological versus technical replication
- The data obtained should be provided and sufficient information should be provided to indicate the number of independent biological and/or technical replicates
- If you encountered any outliers, you should describe how these were handled
- Criteria for exclusion/inclusion of data should be clearly stated
- High-throughput sequence data should be uploaded before submission, with a private link for reviewers provided (these are available from both GEO and ArrayExpress)

Please outline where this information can be found within the submission (e.g., sections or figure legends), or explain why this information doesn’t apply to your submission:

The data reproducibility of all experiments was secured by using biological replicates (different mice) and the number of biological replicates used is mentioned in the figure legends as n=x. The information of the kind of replicates is mentioned in the statistical analysis paragraph of the Materials and methods section.

Single mice were excluded in case the results showed technical failure of the experiment. The information about handling of outliers and exclusion of mice is found in the statistical analysis paragraph of the Materials and methods section.

No high-throughput sequence data are presented in this study.

**Statistical reporting**

- Statistical analysis methods should be described and justified
- Raw data should be presented in figures whenever informative to do so (typically when N per group is less than 10)
- For each experiment, you should identify the statistical tests used, exact values of N, definitions of center, methods of multiple test correction, and dispersion and precision measures (e.g., mean, median, SD, SEM, confidence intervals; and, for the major substantive results, a measure of effect size (e.g., Pearson's r, Cohen's d)
- Report exact p-values wherever possible alongside the summary statistics and 95% confidence intervals. These should be reported for all key questions and not only when the p-value is less than 0.05.

Please outline where this information can be found within the submission (e.g., sections or figure legends), or explain why this information doesn’t apply to your submission:

The general information about the statistical analysis methods is mentioned in the statistical analysis paragraph of the Materials and methods section. Furthermore, for each experiment the information about the statistical tests and the dispersion method used is mentioned in the figure legend.

The results of each mouse are shown in the bar graphs as single data points.

Exact p-values can be found in the source data file.

(For large datasets, or papers with a very large number of statistical tests, you may upload a single table file with tests, Ns, etc., with reference to sections in the manuscript.)

**Group allocation**

- Indicate how samples were allocated into experimental groups (in the case of clinical studies, please specify allocation to treatment method); if randomization was used, please also state if restricted randomization was applied
- Indicate if masking was used during group allocation, data collection and/or data analysis

Please outline where this information can be found within the submission (e.g., sections or figure legends), or explain why this information doesn’t apply to your submission:

For all experiments age-matched wild-type and knock-in mice were compared and for each genotype mice with similar body weights were distributed over the experimental groups that received different diets. During data collection mouse genotype was masked as described in the Mice paragraph of the Material and methods section.

**Additional data files (“source data”)**

- We encourage you to upload relevant additional data files, such as numerical data that are represented as a graph in a figure, or as a summary table
- Where provided, these should be in the most useful format, and they can be uploaded as “Source data” files linked to a main figure or table
- Include model definition files including the full list of parameters used
- Include code used for data analysis (e.g., R, MatLab)
- Avoid stating that data files are “available upon request”

Please indicate the figures or tables for which source data files have been provided:

For all figures except for those that represent a Western blot or microscopic picture source data files are provided. Source data files are provided for: Figure 1A,B,C,D,E,F,G; Figure 1 Supplement 1A, B, C; Figure 2A,B; Figure 3A,C; Figure 4A,C; Figure 5B,D; Figure 5 Supplement 1C,D; Figure 6A,B,C,D; Figure 7A,B; Figure 8B,C,E,F.
